# Mental workload assessment by monitoring brain, heart, and eye with six biomedical modalities during six cognitive tasks

**DOI:** 10.3389/fnrgo.2024.1345507

**Published:** 2024-03-12

**Authors:** Jesse A. Mark, Adrian Curtin, Amanda E. Kraft, Matthias D. Ziegler, Hasan Ayaz

**Affiliations:** ^1^School of Biomedical Engineering, Science, and Health Systems, Drexel University, Philadelphia, PA, United States; ^2^Advanced Technology Laboratories, Lockheed Martin, Arlington, VA, United States; ^3^Department of Psychological and Brain Sciences, College of Arts and Sciences, Drexel University, Philadelphia, PA, United States; ^4^Drexel Solutions Institute, Drexel University, Philadelphia, PA, United States; ^5^A. J. Drexel Autism Institute, Drexel University, Philadelphia, PA, United States; ^6^Department of Family and Community Health, University of Pennsylvania, Philadelphia, PA, United States; ^7^Center for Injury Research and Prevention, Children's Hospital of Philadelphia, Philadelphia, PA, United States

**Keywords:** neuroergonomics, fNIRS, EEG, ECG, EOG, PPG, eye-tracking, multimodal

## Abstract

**Introduction:**

The efficiency and safety of complex high precision human-machine systems such as in aerospace and robotic surgery are closely related to the cognitive readiness, ability to manage workload, and situational awareness of their operators. Accurate assessment of mental workload could help in preventing operator error and allow for pertinent intervention by predicting performance declines that can arise from either work overload or under stimulation. Neuroergonomic approaches based on measures of human body and brain activity collectively can provide sensitive and reliable assessment of human mental workload in complex training and work environments.

**Methods:**

In this study, we developed a new six-cognitive-domain task protocol, coupling it with six biomedical monitoring modalities to concurrently capture performance and cognitive workload correlates across a longitudinal multi-day investigation. Utilizing two distinct modalities for each aspect of cardiac activity (ECG and PPG), ocular activity (EOG and eye-tracking), and brain activity (EEG and fNIRS), 23 participants engaged in four sessions over 4 weeks, performing tasks associated with working memory, vigilance, risk assessment, shifting attention, situation awareness, and inhibitory control.

**Results:**

The results revealed varying levels of sensitivity to workload within each modality. While certain measures exhibited consistency across tasks, neuroimaging modalities, in particular, unveiled meaningful differences between task conditions and cognitive domains.

**Discussion:**

This is the first comprehensive comparison of these six brain-body measures across multiple days and cognitive domains. The findings underscore the potential of wearable brain and body sensing methods for evaluating mental workload. Such comprehensive neuroergonomic assessment can inform development of next generation neuroadaptive interfaces and training approaches for more efficient human-machine interaction and operator skill acquisition.

## 1 Introduction

Human performance on any type of goal or task is related to the amount of cognitive workload that is required to be proficient at completing it. Each person will have their own unique cognitive profile, and be more mentally efficient at performing certain types of tasks (Parasuraman and Jiang, 2012). The amount of effort, as well as activation and therefore required energy expenditure in task-related areas in the brain, changes due to both expertise acquisition and the relative difficulty of the task. While it is possible to track learning via behavioral performance measures, these only provide a measure of the external output of a learner's capabilities and ignore the internal mechanisms that contribute to the results. Therefore, in order to fully track the progress and skill of a learner at any given objective, it is necessary to develop a comprehensive database of the neural and physiological correlates of mental workload, which provide objective and non-invasive measures of the internal state of a learner.

Cognitive workload is a description of the collective external multidimensional demands necessary for an individual to complete a task in proportion with their internal skill level (Hancock and Chignell, 1986; Paas et al., 2003). These external factors place different levels of physical, mental, temporal, and frustration demands on the individual, among others. In order to compensate for these demands and successfully achieve the desired outcome of task performance, a requisite level of skill must be acquired through experience and learning. However, the same performance can potentially be achieved by people of a variety of skill levels. A lower-skilled individual can achieve similar success by applying a high amount of effort whereas a higher-skilled individual may achieve the same result with lower effort. Cognitive workload reflects the amount of effort exerted due to task demands and is amplified by task complexity, individual skill, and experience.

Because cognitive workload is distributed throughout the brain and is an interaction of external and internal factors, there are multiple methods used to measure it. The simplest is to measure behavioral performance and grade it on level of success (John et al., 2002). Although this directly correlates to the output of skill, it is unable to accurately define internal states. The next method is by using subjective surveys such as the NASA-TLX (Hart, 2006). This asks individuals to self-assess their own levels of workload but is inherently lacking due to difficulties inherent in individual's ability to objectively score themselves which may be further marred by memory because it is always given post-task performance. A third method is to use secondary-task performance, which inserts an unrelated task to the primary goal to measure the reserve cognitive capacity (Solovey et al., 2014). The concept is that any mental resources not necessary to be proficient in the main goal will be used by the secondary one, giving a measure of percentage total mental capacity, but the obvious downside is that this is both distracting and puts a lot of strain on the performer. The final method is to use neural and physiological imaging to achieve an objective measure of the inner levels of workload, without putting undo strain on the performer, distracting them, or using unreliable subjective measures.

The assessment of cognitive workload can be applied to any “task” that requires a certain amount of training or practice to master and can refer to any mental or physical profession or hobby, such as flying a plane, performing surgery, operating machinery, overseeing workplace interactions, doing accounting, writing a story, playing an instrument, or more. Each of these real-world tasks may require substantial investment of time, effort, and money to achieve proficiency thereby making any process which lowers the required investment or increases the ease of skill acquisition valuable to both the individual and their workplace. It is important to note that any realistic task as described above is often a complex combination of smaller skills (Wickens et al., 2013). These may involve memory, attention, perceptual motor skills, or multitasking. And each of these domains of cognition may overlap in functional regions of interest in the brain, making it difficult to distinguish which aspects of a complex task are giving a learner trouble. This underlies our goal of finding objective measures of workload that can contribute to accurate tracking of expertise development and be used to optimize personal training. Using a neuroergonomic approach, we seek to uncover methods that can be applied in the real world using currently available technology (Ayaz and Dehais, 2019).

Here we introduce an experimental protocol using a six-task battery focusing on foundational cognitive domains in order to develop a multi-domain multi-modal workload assessment tool. We utilized simple domain-specific cognitive tasks in order to profile work-load contributions associated with each of the following domains: Working Memory (Owen et al., 2005; McKendrick et al., 2014), Vigilance (attention) (Shalev et al., 2011), Risk Assessment (Aklin et al., 2005; Crowley et al., 2006), Shifting Attention (multitasking) (Hagen et al., 2014), Situation Awareness (Endsley, 1988; Wickens, 2002), and Inhibitory Control (Logan et al., 2014; Rodrigo et al., 2014). Together these six domains cover a broad range of basic cognitive components necessary in realistic general task performance.

Within each task, it is also important to assess neurophysiological correlates of multiple levels of workload, representing high and low workload conditions, using hard and easy levels of difficulty respectively. Depending on the current level of skill of a learner, both versions may be easy for someone of high skill, hard for someone of low skill, or one each for a learner of average skill. Over the course of four sessions spanning 1 month, during which each of the six tasks will be presented during three total sessions, we expect participants to increase their skill level, resulting in improved performance and a change in their brain and body workload measures. We also expect to find potentially differential relationships, or interactions, between the task difficulty and session. Based on these measures, we will be able to determine the ideal difficulty of each task to enhance the speed and ease of learning at each stage of expertise within the constraints of our task battery.

During each experimental session, participants were outfitted with a suite of six brain and body sensors to monitor correlates of cognitive workload (Parasuraman and Wilson, 2008; Ayaz et al., 2010b, 2012, 2013; Mehta and Parasuraman, 2013). For neuroimaging, these included functional near-infrared spectroscopy (fNIRS) and electroencephalogram (EEG). Two modalities were used to monitor heart activity, including electrocardiogram (ECG) and photoplethysmography (PPG), and two were used to monitor eye movement activity, electrooculogram (EOG) and eye-tracking. The combination of neuroimaging and peripheral measures helps provide a composite perspective constructed from the central and peripheral nervous systems. These two systems operate in an interrelated fashion that has been overlooked in typical studies focusing on one or the other. These central and peripheral nervous system measures are described in more detail in the methods section below, but each adds a unique contribution to neuro and physiological measurements of cognitive workload correlates.

A single neuroimaging measure can provide useful information on the mental state and inner mechanics of the brain at work, and as different modalities have different advantages and disadvantages, multiple imaging modalities combined is expected to deliver even more detailed information by utilizing the best aspects of each. Therefore, understanding the complementary and shared information in biosignals such as fNIRS, EEG, ECG and other physiological modalities is a long-standing interest (Fazli et al., 2012; Durantin et al., 2014; Putze et al., 2014; Buccino et al., 2016; von Lühmann et al., 2016; Ahn and Jun, 2017; Banville et al., 2017; Chiarelli et al., 2017). In this experiment, we created a new six cognitive domain task protocol and incorporated six biomedical monitoring modalities to simultaneously record performance and correlates of cognitive workload over a longitudinal multi-day study. Our goal was to elucidate the changes in brain and body measurements between high and low workload conditions and over several days/sessions of training as well as directly compare the explained variance of these changes across modalities. This comprehensive workload assessment utilizing both neuroimaging and physiological monitoring can inform the development of next generation neuroadaptive technologies and new training approaches for more efficient skill acquisition.

## 2 Methods

### 2.1 Participants

Twenty-three participants between the ages of 18–48 (16 females, mean age 23 years) volunteered for the study. All subjects confirmed via survey given in person that they met the eligibility requirements of being right-handed with vision correctable to 20/20, did not have a history of brain injury or psychological disorder, were not on medication affecting brain activity, and were United States citizens or permanent residents. Prior to the study all participants signed consent forms approved by the Institutional Review Board of Drexel University.

### 2.2 Protocol

**Figure 1:**
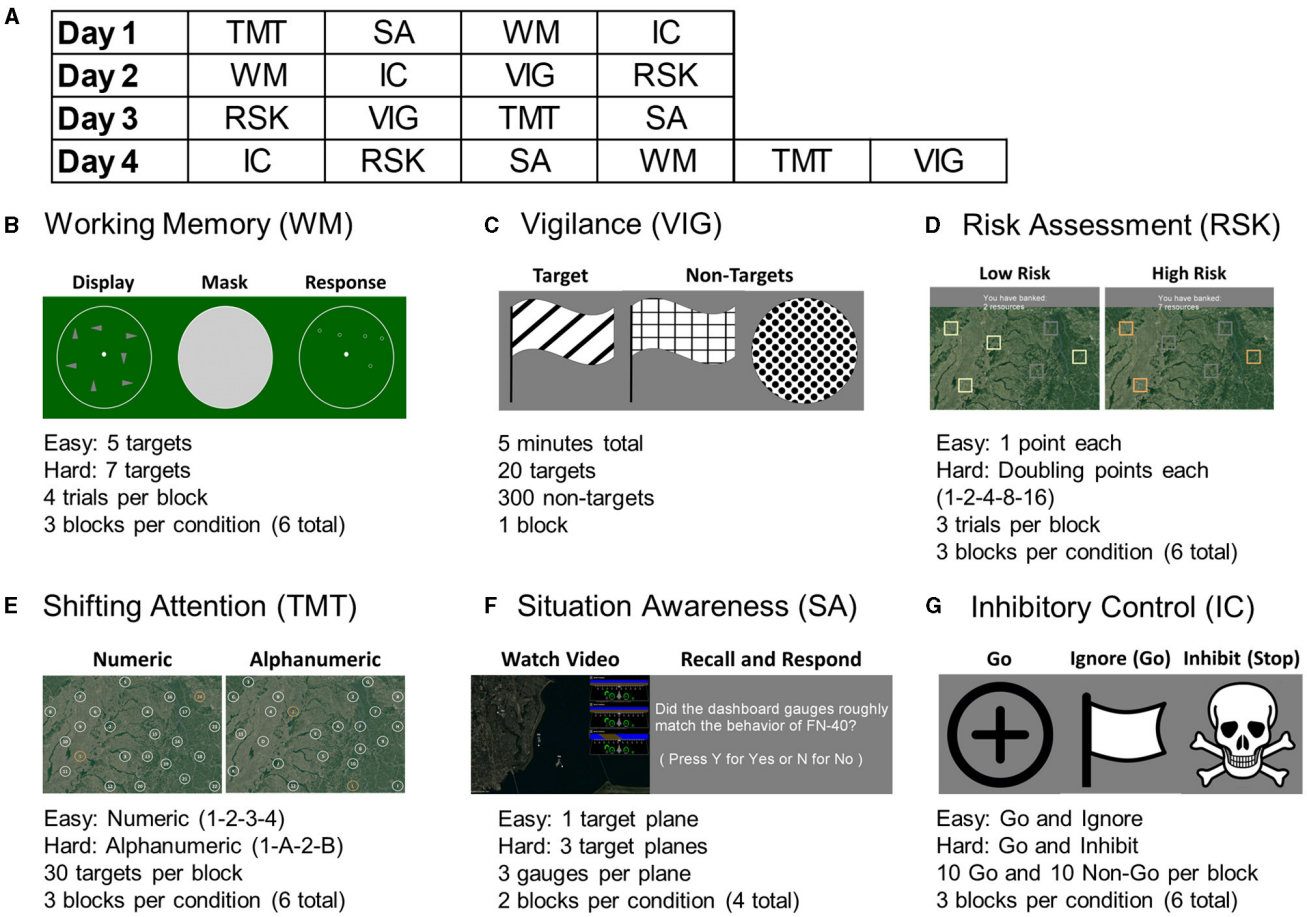
**(A)** An example of the pseudo-random and balanced task ordering over 4 days, with four tasks during the first three and all six tasks on the final day. Each task took 5–8 minutes to complete, with short breaks between. Screenshots and summaries from each task are displayed for **(B)** Working Memory, **(C)** Vigilance, **(D)** Risk Assessment, **(E)** Shifting Attention (trail making task), **(F)** Situation Awareness, and **(G)** Inhibitory Control.

The experiment was performed over four sessions, once a week for 4 weeks, each lasting between 60 and 90 min. Participants were seated upright in front of a computer with a standard mouse and keyboard one meter away from the monitor. They were fitted with an fNIR Devices Model 1200 (fNIR Devices, LLC, Potomac, MD) headband over the forehead, a Cognionics HD-72 dry electrode cap (Cognionics, Inc, San Diego, CA), and a Cognionics extension providing sticky electrodes for the ECG (3 electrodes), EOG (4 electrodes), and PPG ear clip (described in more detail in Section 2.4). Eye tracking was calibrated using the Smart Eye Aurora system (Smart Eye, AB, Gothenburg, Sweden) recording gaze location and pupil diameter. Time synchronization between each of the six modalities (over three separate devices) was performed programmatically with a custom Python script that sent markers to each data collection system simultaneously via local area network. Task block markers at the start and end of each block, as well as at each significant stimuli, were sent simultaneously to all recording software. The six task protocol was implemented using the Python based PsychoPy application (Peirce et al., 2019). Task performance was preceded by instructions and practice trials for each difficulty condition where subjects could familiarize themselves with the procedure and ask clarifying questions. Each task was designed to take 5–8 min to complete. During each of the first three sessions, participants performed four of the six tasks (selected in a counter-balanced order) and performed all six tasks in the final session, for a total of three sessions per task (Figure 1A).

### 2.3 Task battery

#### 2.3.1 Working Memory

The working memory task was a modification of the spatial working memory task (Owen et al., 2005; McKendrick et al., 2014). A blank “radar” was displayed for 250 ms followed by 1 s stimuli of either five or seven targets, representing easy and hard conditions, which were to be memorized. Three seconds of a static noise image were displayed to prevent ocular “burn-in” memorization, followed by a 15 second response window where participants clicked as near as possible to the target locations. This was followed by 750 ms of break. Four trials of each difficulty were presented per block, and three blocks of each condition were presented per session (Figure 1B).

#### 2.3.2 Vigilance

Vigilance was evaluated using an adaptation of the conjunctive continuous performance task (Shalev et al., 2011). A series of shapes with varying fill patterns were displayed over 5 min, with targets that required a response of clicking a key, and non-targets of differing shapes and fill patterns. Each stimulus was presented for 100 ms with a 750 ± 250 ms variable interstimulus interval. Targets requiring a response were 1/16 of the total 320 stimuli. The ratio of targets to nontargets was balanced over eight evenly split segments. The first four were taken as low workload and the latter four were taken as high workload conditions (Figure 1C).

#### 2.3.3 Risk Assessment

Decision-making was evaluated using a task based on the Balloon Analog Risk Task (BART) (Aklin et al., 2005; Crowley et al., 2006). The goal of this task was to collect as many resources as possible without “crashing” a virtual search vehicle. Six total targets were displayed to be clicked in any order with the goal of collecting as many “points” as possible by the end. In the easy condition, each block provided one additional point, and there was a low chance of crashing and losing all of that round's points. In the hard condition, each block doubled in value (1, 2, 4, 8, 16), but there was a higher chance of crashing. Participants were given 5 s to make a decision, with 1.5 s feedback after a click (current points or if crashed), and 5 s between trials. Three trials of each condition were conducted per block, with three blocks of each condition per session (Figure 1D).

#### 2.3.4 Shifting Attention

A digitized trail making test (TMT) with three blocks each of two difficulty conditions (Hagen et al., 2014; Müller et al., 2014). Participants used the mouse to click randomly placed circles in numeric (1-2-3-4) order for the easy condition and alternating alphanumeric (1-A-2-B) in the hard condition. The hard condition represented shifting attention between the two mental lists of numbers and letters. Each block was 30 s maximum, and there were three blocks of each difficulty per session (Figure 1E).

#### 2.3.5 Situation Awareness

Subjects viewed 30 s prerecorded videos of a top-down aircraft flight simulation of either one plane (easy condition) or three planes (hard condition) flying various paths (Endsley, 1988; Wickens, 2002). Each plane had a corresponding dashboard visible with three gauges representing heading, speed, and fuel level. The goal of this task was to determine which, if any, of these dashboard levels on the side of the screen did not accurately match the actual condition of the planes in a series of questions presented after each recording. Each difficulty was presented for two blocks each per session (Figure 1F).

#### 2.3.6 Inhibitory Control

A modified go-no go task with the “go” (easy) condition called “ignore” and the “stop” (hard) condition called “inhibit” (Logan et al., 2014; Rodrigo et al., 2014). In each block a total of 20 “go” stimuli were presented for 500 ms with 1,000 ± 200 ms interstimulus interval, and participants were told to click a key in response. In half of these trials, after 150 ms the go target changed to a flag in the easy condition, which participants were told to ignore, or a skull and cross in the hard condition, which participants had to inhibit their response. Participants were instructed specifically to start a response as soon as the go signal was visible, and to not “wait and see”. Three blocks of each difficulty were presented each session (Figure 1G).

### 2.4 Sensors and signal processing

**Figure 2:**
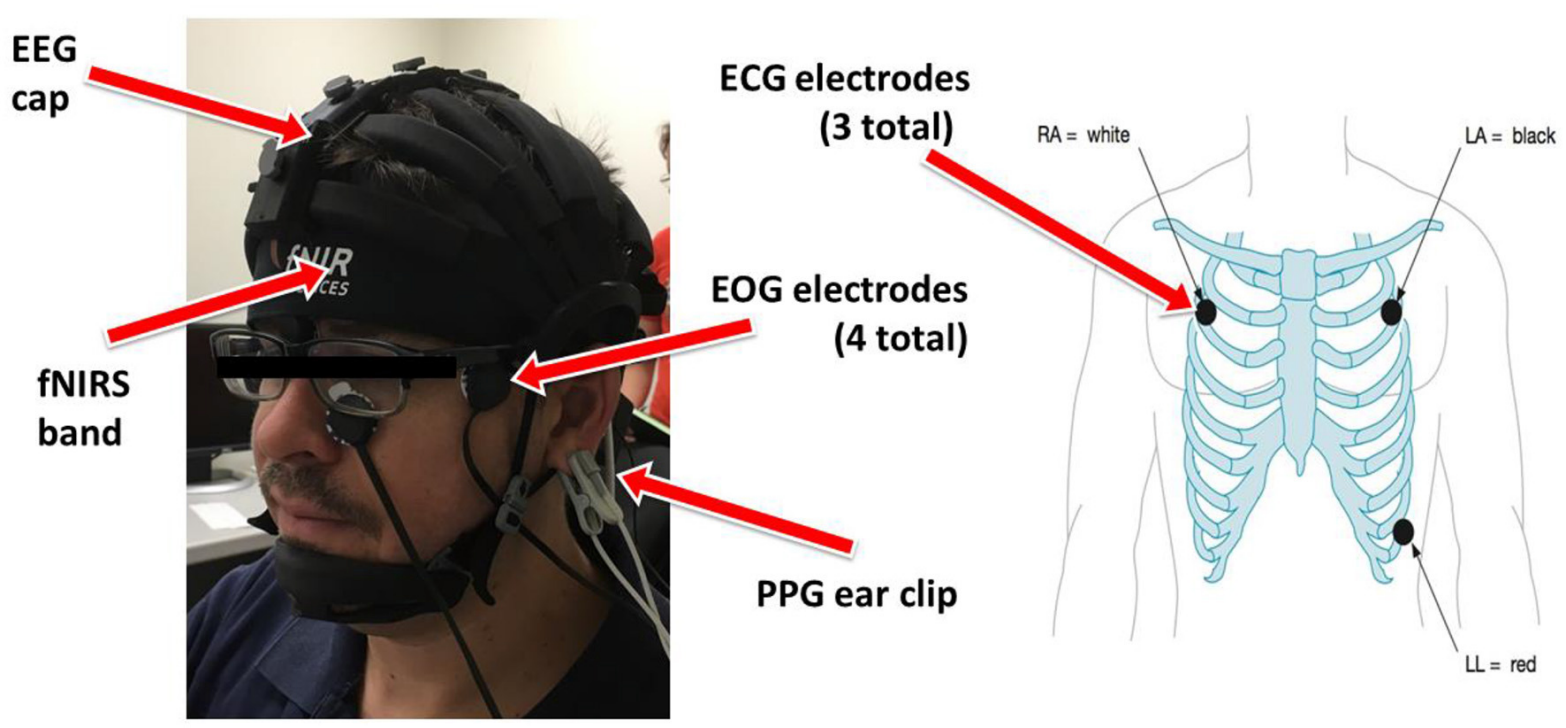
Experimental setup detailing the locations of each modality. **Left**: EEG, fNIRS, PPG, EOG placement on participant's head. **Right**: ECG placement on participants chest.

One fNIRS headband and one wireless EEG system with an extension box allowing for simultaneous ECG, PPG, and EOG were placed on each participant at the start of each session as can be seen in Figure 2. The eye-tracking system was installed below the experimental computer monitor and calibrated prior to each experiment.

#### 2.4.1 Functional near-infrared spectroscopy

Functional near-infrared spectroscopy uses light in the wavelengths of the near-infrared range (in the optical window of human tissue from 700 nm to 900 nm) to measure changes in the local concentration levels of oxygenated- and deoxygenated-hemoglobin in the cortical tissue (Ayaz et al., 2022). This hemodynamic response correlates with the specific neuronal activity of the measured brain areas via neurovascular coupling and provides brain activity change information on the relative changes of oxygenated blood concentration (Yücel et al., 2021; Ayaz et al., 2022). Furthermore, fNIRS systems can be built miniaturized and are suitable for out-of-lab and even ambulatory measurements (Piper et al., 2014; McKendrick et al., 2016; Quaresima and Ferrari, 2016; Balardin et al., 2017; Curtin and Ayaz, 2018; Pinti et al., 2018; von Lühmann et al., 2021). In our experiment, the fNIR Devices Model 1200 was used to record prefrontal cortical hemodynamics (Ayaz et al., 2013). We recorded from 16 optode locations at a rate of 2 Hz. Raw light intensity taken at 730 and 850 nm was filtered with a low pass FIR filter (Hamming window, order 20 and cutoff frequency 0.1 Hz) and a sliding window motion artifact rejection (SMAR) algorithm (Ayaz et al., 2010a) in Matlab, and then processed using the modified Beer-Lambert Law into oxygenated and deoxygenated hemoglobin values. The oxygenated hemoglobin (HbO), deoxygenated hemoglobin (HbR), oxygenation (Oxy = HbO – HbR), and total blood (HbT = HbO + HbR) values were block processed for the mean, slope, peak value, time-to-peak, and sum of changes for each task and condition.

#### 2.4.2 Electroencephalogram

EEG measures highly temporally localized electrical activity of neuron groups in the cortex via electrodes placed over the scalp. Its strength is in determining the precise timing of brain reactions to stimuli and thoughts, as well as provide higher order measures of brain waves in the alpha, beta, delta, and theta frequency bands of activity, as well as combinations of these (Gruzelier, 2014). EEG systems have been undergoing decades of development, and currently many types of systems such as active vs. passive and dry vs. wet electrodes as well as battery-operated and high density shielded stationary systems exist (Zander et al., 2017; Marini et al., 2019). There have been many developments on EEG methodology toward enabling mobile brain imaging in more naturalistic settings (De Vos and Debener, 2014; Gramann et al., 2014; Wascher et al., 2021). In our experiment, the Cognionics HD-72 dry electrode EEG was used to record full head neuronal measures. Data was collected from 32 electrodes at 500 Hz after checking for impedance and processed using a Butterworth order 8 notch filter at 60 Hz, followed by a Butterworth order 7 bandpass filter between 1 and 59 Hz. EEGLAB functions for independent component analysis (ICA) were used to remove eye movement and muscle motion artifacts, followed by Automatic Subspace Reconstruction (ASR) to clean noise and estimate missing segments (Delorme et al., 2011; Mullen et al., 2013). Continuous band power calculations for each channel were done using Welch's power spectral density of the EEG signal with a moving window of 2 s. Power spectra were divided into delta (1–4 Hz), theta (4–8 Hz), alpha (8–13 Hz), beta (13–30 Hz) and gamma (>30 Hz) bands. In addition, power band combinations including the engagement ratio beta/(alpha+theta), theta/alpha, theta/beta, and (theta+alpha)/(beta+alpha) were analyzed for workload assessment (Cao et al., 2014; Ismail and Karwowski, 2020).

#### 2.4.3 Electrocardiogram

Heart activity is affected by mental effort and environmental stressors (Shaffer and Ginsberg, 2017). This includes not just heart rate and heart rate variability, but the shape of the signal and other temporal measures. In this experiment, heart activity was recorded from three electrodes via an extension to the Cognionics headset. The Matlab extension HEPLAB (Perakakis, 2021) with default settings was used to process ECG data, and was manually corrected afterwards. Heart rate, heart rate variability (standard deviation and root mean squared), low frequency power (0.04–0.15 Hz, absolute and relative), high frequency power (0.15–0.4 Hz, absolute and relative), and LF/HF ratio measures of workload were processed using Matlab (Roscoe, 1992).

#### 2.4.4 Photoplethysmography

Photoplethysmography (PPG) is a versatile modality for measuring blood flow and can be used to supplement ECG and add additional factors to heart monitoring. Here, systemic blood flow was recorded from an optical ear clip extension to the Cognionics headset. PPG data was processed in Matlab with a Butterworth bandpass filter (0.1–10 Hz, order 7) and the find peaks function with custom correction for ignoring false peaks, plus manual correction, was used to extract data (Shaffer and Ginsberg, 2017). The same measures as ECG were extracted (heart rate, HRV, LF, HF, LF/HF ratio) in addition to average width and average peak of pulses.

#### 2.4.5 Electrooculogram

Blinks, saccades, and eye movements correlate with mental workload (Marquart et al., 2015). Using a distinct EOG system separate from EEG electrodes allows for cleaner signal that is not contaminated by other information. In the experiment, eye movements were recorded from four electrodes via an extension to the Cognionics headset, two placed above and below the left eye, and two played on the outside of both eyes, to separately record vertical and horizontal movement. The raw data was processed using an implementation of Behrens et al.'s (2010) improved detection of saccades algorithm. This provided workload correlates of saccade velocity, duration, and amplitude.

#### 2.4.6 Eye-tracking

Saccade velocity, fixations, pupil diameter, and their variations are known correlates of cognitive workload (Ahlstrom and Friedman-Berg, 2006). Eye tracking can also provide a more accurate assessment of precise gaze location, whereas EOG may be able to measure smaller, subtler movements of the eye. In this experiment, the Smart Eye Aurora recorded eye gaze and pupil diameter at 60 Hz and was processed using OGAMA (Open Gaze and Mouse Analyzer) software (Voßkühler et al., 2008). Pupil diameter, saccade velocity, saccade length, number of fixations, average fixation duration, fixation rate, and the fixation to saccade ratio were calculated as correlates of cognitive workload (Ahlstrom and Friedman-Berg, 2006).

### 2.5 Statistical processing

Linear mixed models were applied to determine the significant effects of task session (1, 2, and 3), condition (easy and hard), and their interaction on each task-specific performance measure and a comprehensive range of block processed correlates of cognitive workload for each modality (fNIRS, EEG, ECG, PPG, EOG, and eye-tracking). These models included subject as a random effect to account for inter-subject differences, particularly within the neuroimaging modalities. The diagonal covariance pattern and restricted maximum likelihood (REML) methods were used. Significance was assessed by F-values and corresponding *p*-values for each measurement. The Benjamini-Hochberg false discovery rate (FDR) correction was applied to fNIRS and EEG results across optodes and electrodes respectively for each processed data type with α = 0.05. Effect sizes were calculated using the partial eta-squared values.

Principal components analysis (PCA) was used to determine the percentage of explained variance of all of the data combined across sessions and conditions. For each task, all principal components with eigenvalues above 1 were isolated, and the coefficients and percentage of the total for each modality was calculated. The procedure was run 30 times with different starting seeds to achieve a more accurate estimate of the true variance, as well as the number of components. PCA provides an equal comparison method between modalities that does not rely on linear model parameters.

## 3 Results

Due to the scale of the analyses performed in this study, only the most significant and relevant results will be presented in the main body of this publication for each task and modality. Entire list of results from all modalities, features, task conditions with significance and effect sizes are listed in Supplementary Table. The standardized methodology for selecting data for the following tables is: Maximum of five points per modality (performance, fNIRS, EEG, ECG, PPG, EOG, Eye-tracking); EEG and fNIRS must be significant after FDR correction; begin with the highest effect size (partial eta-squared); have at least one row for each significant factor, if available (session, condition, interaction). The remainder of the full statistics can be found in the Supplementary material. For the Figures, fNIRS and EEG results are presented for each main factor and interaction, and performance and physiological measures are presented in combination. For LMM and PCA analyses, we used 320 fNIRS measures (160 in PCA), 288 EEG measures (224 in PCA), 8 ECG measures, 10 PPG measures, 3 EOG measures, and 8 eye-tracking measures (number of performance measures differed between task from 3 to 6 and were not included in PCA).

### 3.1 Working Memory

**Figure 3:**
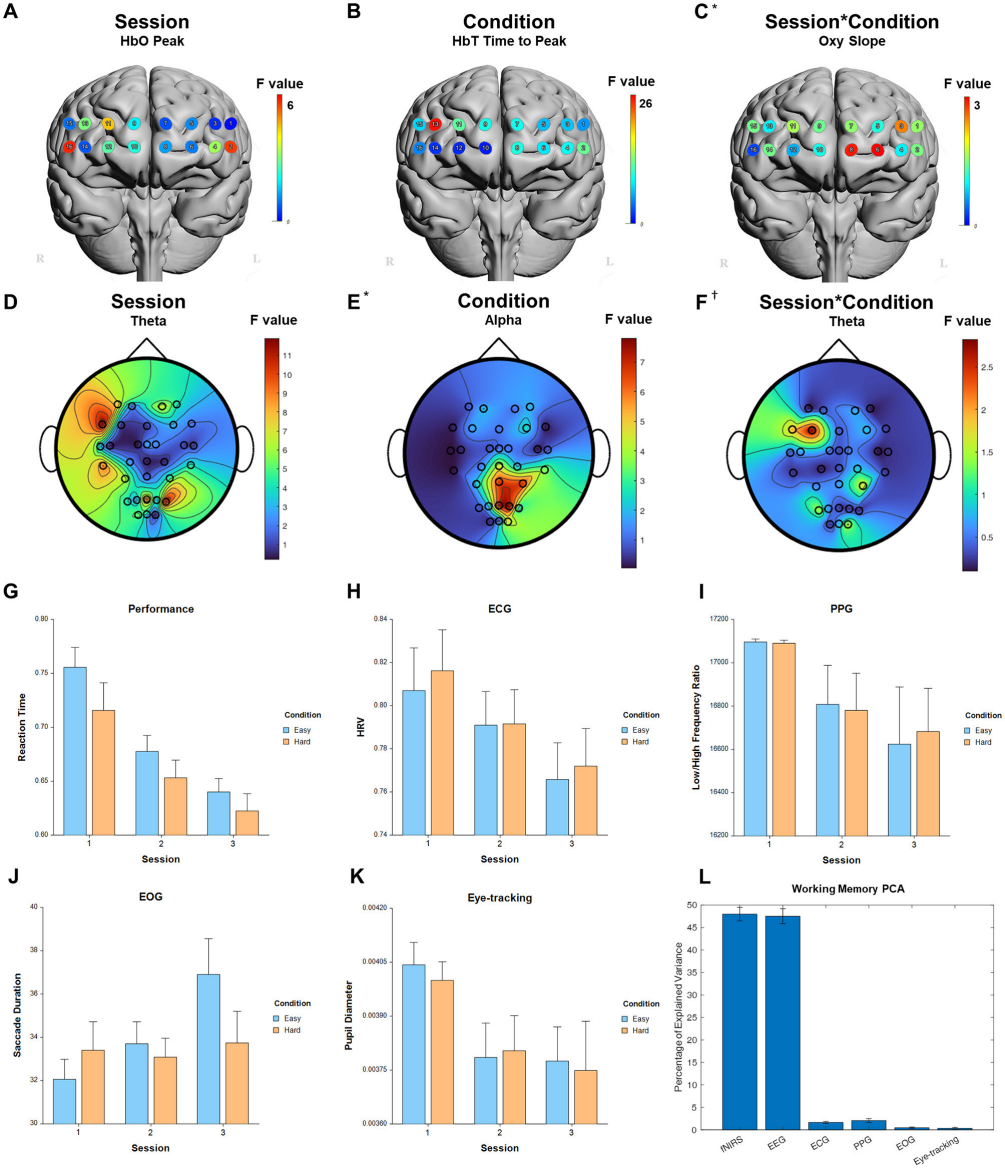
Selected results for Working Memory task. **(A–C)** LMM *F*-values for fNIRS across prefrontal cortex for the fixed factors of Session, Condition, and Interaction. **(D–F)** LMM *F*-values for EEG across whole brain for the fixed factors of Session, Condition, and Interaction. **(G)** Performance measure across session and condition. **(H)** ECG measure. **(I)** PPG measure. **(J)** EOG measure. **(K)** Eye-tracking measure. **(L)** PCA percentage of explained variance. ^*^Significant before FDR correction. ^†^Not significant.

For the fixed factor of session, significant measures included: 1 performance, 54 fNIRS (29 FDR-corrected), 223 EEG (214 FDR-corrected), 5 ECG, 4 PPG, 3 EOG, and 1 eye-tracking. For the fixed factor of condition, significant measures included: 4 performance, 41 fNIRS (22 FDR-corrected), and 10 EEG (0 FDR-corrected). For session and condition interaction, significant measures included: 4 fNIRS (0 FDR-corrected). PCA analysis revealed an average of 16.7 PCs with eigenvalues greater than 1, explaining 72.4% of the total variance. Of this, the following percentage of variance explained by each modality is: 48.0% fNIRS, 47.5% EEG, 1.6% ECG, 2.1% PPG, 0.5% EOG, and 0.3% eye-tracking. Selected results are presented in Figure 3, Table 1.

**Table 1:** Selected results of LMM processing for performance and each modality for Working Memory.

**Modality**	**Measure**	**Factor**	**F-ratio**		***p*-value**	**Partial η^2^**
**Performance**						
	Reaction time	Session	39.59	_2/380.4_	0.000001	0.172
	Reaction time	Condition	7.81	_1/380_	0.005468	0.020
	Accuracy	Condition	125.63	_1/374.9_	0.000001	0.251
**fNIRS**						
	HbO 02 peak	Session	5.54	_2/381.2_	0.004245	0.028
	HbO 16 peak	Session	5.73	_2/378.1_	0.003529	0.029
	HbT 02 slope	Session	12.38	_2/368.4_	0.000006	0.063
	HbT 11 time-to-peak	Condition	12.02	_1/379.7_	0.000588	0.031
	HbT 13 time-to-peak	Condition	25.41	_1/380.1_	0.000001	0.063
**EEG**						
	Theta CPP3h	Session	6.30	_2/34.6_	0.004635	0.267
	Alpha PO1	Session	47.70	_2/203.8_	0.000001	0.319
	Engagement CPPz	Session	52.16	_2/138.3_	0.000001	0.430
	Theta/Alpha FFC5h	Session	20.49	_2/206.1_	0.000001	0.166
	Theta + Alpha/Beta + Alpha FCC6h	Session	20.26	_2/209.3_	0.000001	0.162
**ECG**						
	Heartrate	Session	14.28	_2/324_	0.000001	0.081
	HRV (rms)	Session	15.29	_2/321.7_	0.000001	0.087
	Low frequency (abs)	Session	10.25	_2/322.8_	0.000048	0.060
**PPG**						
	Low frequency (rel)	Session	15.14	_2/4_	0.013614	0.883
	High frequency (rel)	Session	15.14	_2/4_	0.013614	0.883
	Low/high ratio	Session	8.97	_2/325.9_	0.000161	0.052
**EOG**						
	Peak saccade velocity	Session	7.61	_2/4_	0.043346	0.792
	Saccade duration	Session	4.36	_2/326.9_	0.013575	0.026
	Saccade amplitude	Session	3.57	_2/331.1_	0.029288	0.021
**Eye-tracking**						
	Pupil diameter	Session	12.83	_2/4_	0.018185	0.865

fNIRS and EEG have been FDR-corrected. A lack of results for a factor (i.e., Condition, Interaction) indicates no significant results.

### 3.2 Vigilance

**Figure 4:**
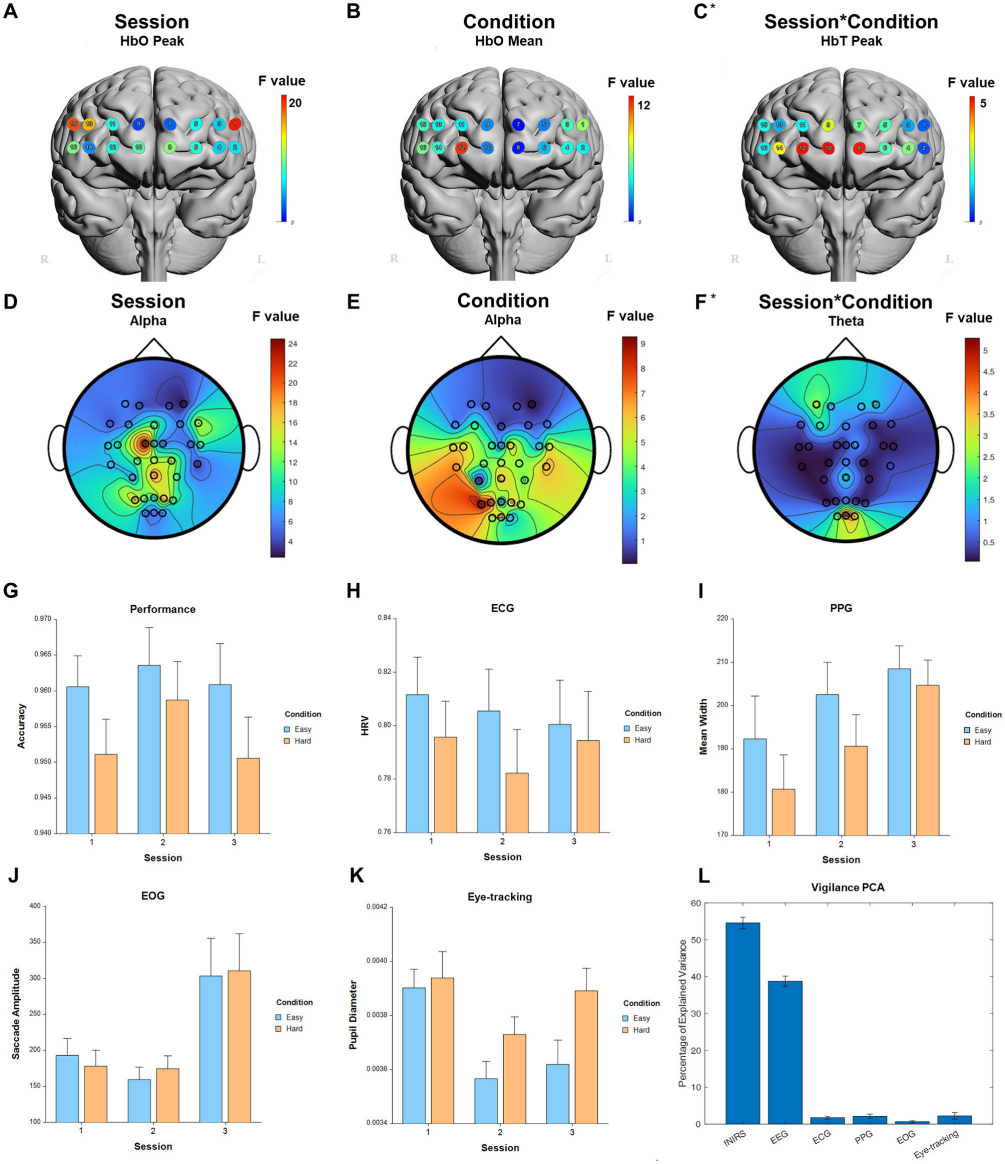
Selected results for Vigilance task. **(A–C)** LMM *F*-values for fNIRS across prefrontal cortex for the fixed factors of Session, Condition, and Interaction. **(D–F)** LMM *F*-values for EEG across whole brain for the fixed factors of Session, Condition, and Interaction. **(G)** Performance measure across session and condition. **(H)** ECG measure. **(I)** PPG measure. **(J)** EOG measure. **(K)** Eye-tracking measure. **(L)** PCA percentage of explained variance. *Significant before FDR correction.

For the fixed factor of session, significant measures included: 1 performance, 72 fNIRS (53 FDR-corrected), 246 EEG (237 FDR-corrected), 6 ECG, 7 PPG, 2 EOG, and 4 eye-tracking. For the fixed factor of condition, significant measures included: 4 performance, 38 fNIRS (2 FDR-corrected), 44 EEG (1 FDR-corrected), 1 ECG, 2 PPG, and 7 eye-tracking. For session and condition interaction, significant measures included: 16 fNIRS (3 FDR-corrected) and 14 EEG (0 FDR-corrected). PCA analysis revealed an average of 18.6 PCs with eigenvalues >1, explaining 71.8% of the total variance. Of this, the following percentage of variance explained by each modality is: 54.4% fNIRS, 38.8% EEG, 1.7% ECG, 2.0% PPG, 0.7% EOG, and 2.3% eye-tracking. Selected results are presented in Figure 4, Table 2.

**Table 2:** Selected results of LMM processing for performance and each modality for Vigilance.

**Modality**	**Measure**	**Factor**	**F-ratio**		***p*-value**	**Partial η^2^**
**Performance**						
	Reaction time	Session	12.45	_2/524_	0.000005	0.045
	Reaction time	Condition	22.51	_1/524_	0.000003	0.041
	Accuracy	Condition	6.39	_1/524_	0.011789	0.012
	True positive	Condition	11.97	_1/524_	0.000586	0.022
**fNIRS**						
	HbO 01 peak	Session	19.42	_2/524_	0.000001	0.069
	HbO 15 peak	Session	18.07	_2/523_	0.000001	0.065
	HbO 12 mean	Condition	11.25	_1/523_	0.000854	0.021
	HbT 10 peak	Session^*^Condition	4.94	_2/498.9_	0.007503	0.019
	HbT 12 peak	Session^*^Condition	4.87	_2/515_	0.008064	0.019
**EEG**						
	Theta CPP3h	Session	33.76	_1/27_	0.000004	0.556
	Alpha CPPz	Session	18.15	_2/208.4_	0.000001	0.148
	Engagement FCC5h	Session	45.09	_2/321.8_	0.000001	0.219
	Theta + Alpha/Beta + Alpha CPP3h	Session	54.29	_1/26.9_	0.000001	0.669
	Delta FCC6h	Condition	10.24	_1/309.1_	0.001515	0.032
**ECG**						
	Heartrate	Session	6.54	_2/448.1_	0.001583	0.028
	Low frequency (rel)	Session	10.13	_2/4_	0.027182	0.835
	High frequency (rel)	Session	10.13	_2/4_	0.027182	0.835
	Low/High ratio	Session	9.12	_2/450.3_	0.000132	0.039
	HRV (rms)	Condition	6.28	_1/444_	0.012573	0.014
**PPG**						
	Heartrate	Session	7.20	_2/439.1_	0.000842	0.032
	Mean width	Session	4.09	_2/438.9_	0.017382	0.018
	HRV (rms)	Session	5.82	_2/439.8_	0.003215	0.026
	Heartrate	Condition	4.43	_1/436_	0.035813	0.010
	Mean width	Condition	7.32	_1/436_	0.007087	0.017
**EOG**						
	Peak saccade velocity	Session	15.46	_2/433.5_	0.000001	0.067
	Saccade amplitude	Session	16.88	_2/432.6_	0.000001	0.072
**Eye-tracking**						
	Pupil diameter	Session	13.89	_2/4_	0.015843	0.874
	Saccade velocity	Session	3.70	_2/264.3_	0.025965	0.027
	Fixation count	Session	4.96	_2/256.5_	0.007693	0.037
	Pupil diameter	Condition	15.35	_1/4_	0.017287	0.793
	Mean fixation duration	Condition	29.82	_1/248.9_	0.000001	0.107

fNIRS and EEG have been FDR-corrected. A lack of results for a factor (i.e., Condition, Interaction) indicates no significant results.

### 3.3 Risk Assessment

**Figure 5:**
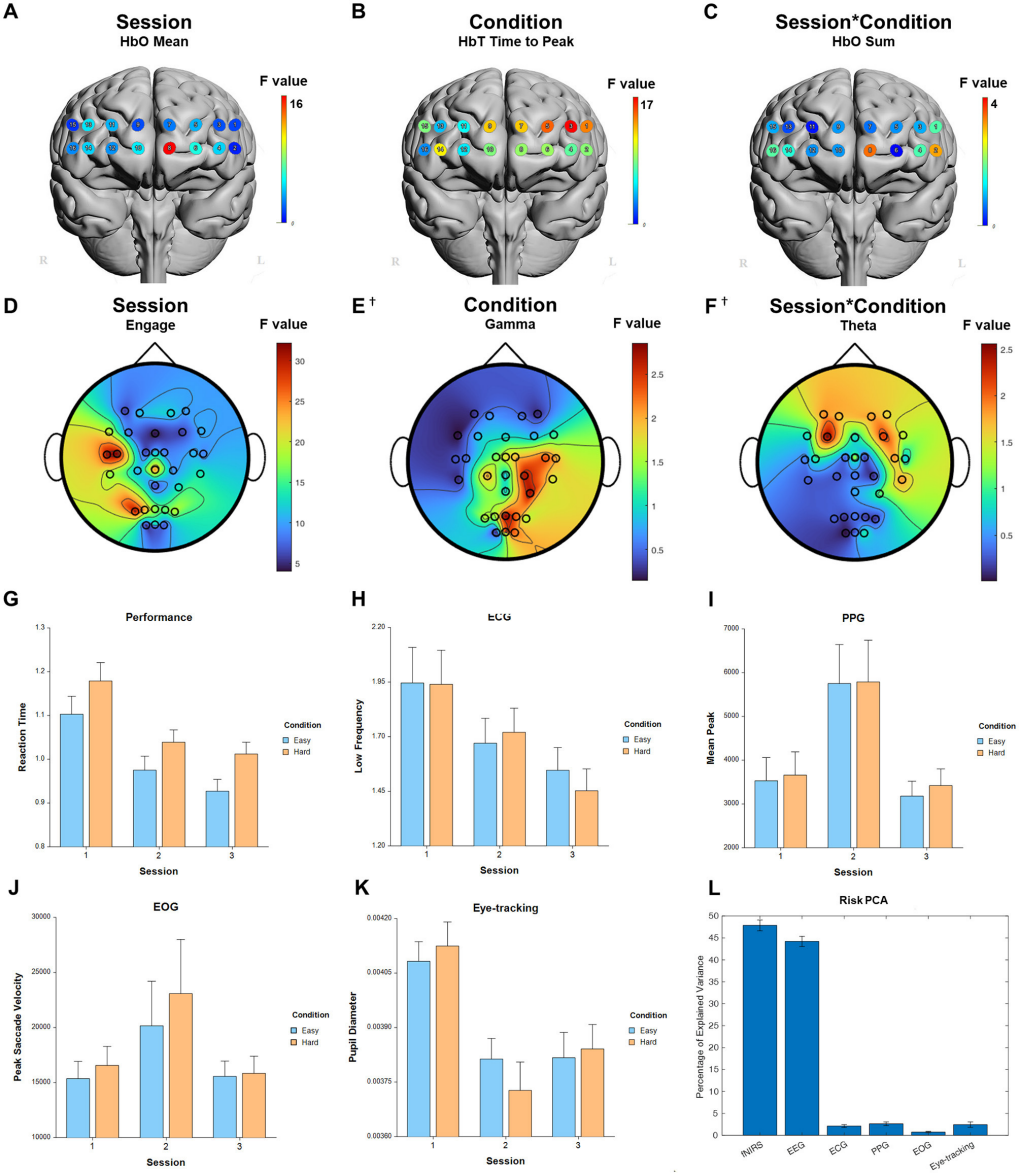
Selected results for Risk Assessment task. **(A–C)** LMM *F*-values for fNIRS across prefrontal cortex for the fixed factors of Session, Condition, and Interaction. **(D–F)** LMM *F*-values for EEG across whole brain for the fixed factors of Session, Condition, and Interaction. **(G)** Performance measure across session and condition. **(H)** ECG measure. **(I)** PPG measure. **(J)** EOG measure. **(K)** Eye-tracking measure. **(L)** PCA percentage of explained variance. ^†^Not significant.

For the main factor of session, significant measures included: 1 performance, 96 fNIRS (58 FDR-corrected), 229 EEG (225 FDR-corrected), 3 ECG, 9 PPG, 2 EOG, and 4 eye-tracking. For the fixed factor of condition, significant measures included: 5 performance, 36 fNIRS (31 FDR-corrected), and 1 EEG (0 FDR-corrected). For session and condition interaction, significant measures included: 11 fNIRS (0 FDR-corrected). PCA analysis revealed an average of 16.4 PCs with eigenvalues greater than 1, explaining 71.2% of the total variance. Of this, the following percentage of variance explained by each modality is: 47.5% fNIRS, 44.8% EEG, 2.1% ECG, 2.6% PPG, 0.7% EOG, and 2.4% eye-tracking. Selected results are presented in Figure 5, Table 3.

**Table 3:** Selected results of LMM processing for performance and each modality for Risk Assessment.

**Modality**	**Measure**	**Factor**	**F-ratio**		***p*-value**	**Partial η^2^**
**Performance**						
	Reaction time	Session	17.57	_2/380.5_	0.000001	0.085
	Reaction time	Condition	10.31	_1/379.6_	0.001437	0.026
	Successful clicks	Condition	332.33	_1/380.3_	0.000001	0.466
**fNIRS**						
	HbO 08 mean	Session	16.16	_2/379.5_	0.000001	0.078
	Oxy 08 mean	Session	12.84	_2/379.4_	0.000004	0.063
	HbT 01 peak	Session	13.94	_2/381.1_	0.000001	0.068
	HbT 03 time-to-peak	Session	6.36	_2/382.1_	0.001917	0.032
	HbT 03 time-to-peak	Condition	17.10	_1/379.8_	0.000044	0.043
**EEG**						
	Theta CPPz	Session	16.47	_2/144.2_	0.000001	0.186
	Alpha PO3	Session	18.40	_2/212.3_	0.000001	0.148
	Engagement FCC3	Session	32.21	_2/197.4_	0.000001	0.246
	Theta/Alpha CPPz	Session	13.62	_2/141.8_	0.000004	0.161
	Theta + Alpha/Beta + Alpha PO1	Session	23.57	_2/227.9_	0.000001	0.171
**ECG**						
	Heartrate	Session	4.50	_2/316.7_	0.011857	0.028
	Low frequency (abs)	Session	9.29	_2/315.7_	0.00012	0.056
	High frequency (abs)	Session	7.47	_2/4_	0.044626	0.789
**PPG**						
	Mean peak	Session	17.72	_2/317.2_	0.000001	0.101
	Low frequency (rel)	Session	8.55	_2/4_	0.035938	0.810
	High frequency (rel)	Session	8.55	_2/4_	0.035938	0.810
**EOG**						
	Peak saccade velocity	Session	9.25	_2/4_	0.031605	0.822
	Saccade amplitude	Session	3.86	_2/315.2_	0.022135	0.024
**Eye-tracking**						
	Pupil diameter	Session	37.10	_2/4_	0.002617	0.949
	Mean fixation duration	Session	6.48	_2/200.6_	0.001874	0.061

fNIRS and EEG have been FDR-corrected. A lack of results for a factor (i.e. Condition, Interaction) indicates no significant results.

### 3.4 Shifting Attention

**Figure 6:**
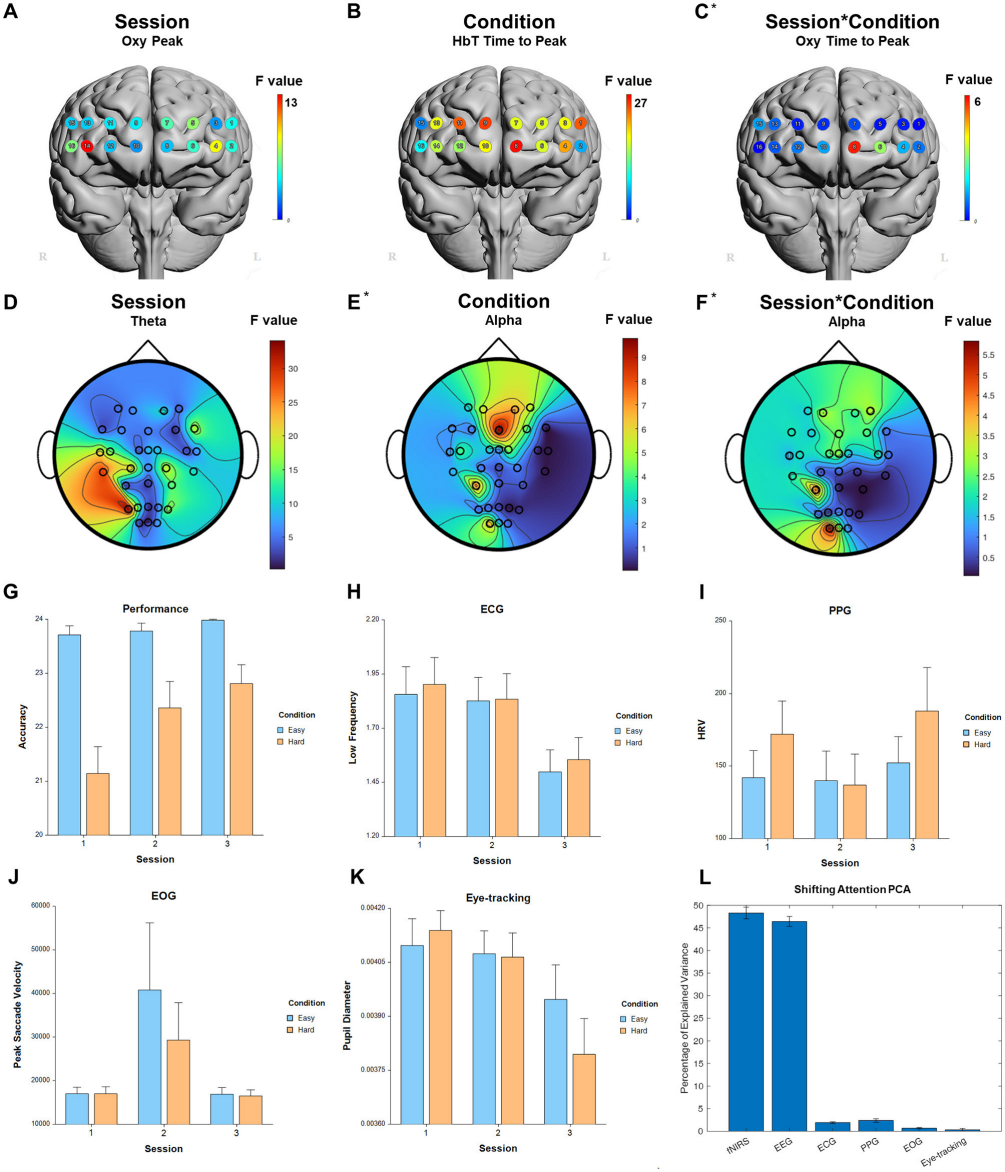
Selected results for Shifting Attention task. **(A–C)** LMM *F*-values for fNIRS across prefrontal cortex for the fixed factors of Session, Condition, and Interaction. **(D–F)** LMM *F*-values for EEG across whole brain for the fixed factors of Session, Condition, and Interaction. **(G)** Performance measure across session and condition. **(H)** ECG measure. **(I)** PPG measure. **(J)** EOG measure. **(K)** Eye-tracking measure. **(L)** PCA percentage of explained variance. *Significant before FDR correction.

For the fixed factor of session, significant measures included: 5 performance, 74 fNIRS (52 FDR-corrected), 218 EEG (208 FDR-corrected), 3 ECG, 3 PPG, 2 EOG, and 2 eye-tracking. For the fixed factor of condition, significant measures included: 5 performance, 47 fNIRS (32 FDR-corrected), 22 EEG (0 FDR-corrected), and 1 ECG. For session and condition interaction, significant measures included: 4 performance, 11 fNIRS (0 FDR-corrected), 6 EEG (0 FDR-corrected), and 1 eye-tracking. PCA analysis revealed an average of 17.7 PCs with eigenvalues greater than 1, explaining 65.9% of the total variance. Of this, the following percentage of variance explained by each modality is: 48.4% fNIRS, 46.4% EEG, 1.9% ECG, 2.3% PPG, 0.7% EOG, and 0.2% eye-tracking. Selected results are presented in Figure 6, Table 4.

**Table 4:** Selected results of LMM processing for performance and each modality for Shifting Attention.

**Modality**	**Measure**	**Factor**	**F-ratio**		***p*-value**	**Partial η^2^**
**Performance**						
	Accuracy	Session	6.33	_2/386_	0.00197	0.032
	Accuracy	Condition	57.52	_1/386_	0.000001	0.130
	Accuracy	Session^*^Condition	3.57	_2/386_	0.029004	0.018
	Reaction time	Session	15.38	_2/386_	0.000001	0.074
	Reaction time	Condition	96.14	_1/386_	0.000001	0.199
	Reaction time	Session^*^Condition	3.10	_2/386_	0.046174	0.016
**fNIRS**						
	HbO 05 peak	Session	8.44	_2/381.6_	0.00026	0.042
	Oxy 14 peak	Session	13.15	_2/379.5_	0.000003	0.065
	Oxy 16 peak	Session	6.56	_2/374.9_	0.001581	0.034
	HbT 08 time-to-peak	Condition	26.61	_1/375.5_	0.000001	0.066
	HbT 09 time-to-peak	Condition	24.97	_1/386_	0.000001	0.061
**EEG**						
	Theta CCP1	Session	21.97	_2/214.6_	0.000001	0.170
	Theta PO3	Session	34.28	_2/216_	0.000001	0.241
	Alpha CPP3h	Session	9.64	_2/15.6_	0.001882	0.553
	Engagement PO3	Session	43.48	_2/214.9_	0.000001	0.288
	Theta/Alpha FFC6h	Session	24.66	_2/244.3_	0.000001	0.168
**ECG**						
	Low frequency (abs)	Session	27.11	_2/356.8_	0.000001	0.132
	High frequency (abs)	Session	7.62	_2/4_	0.043232	0.792
	Low/High ratio	Session	4.20	_2/361.3_	0.015767	0.023
	Heartrate	Condition	5.21	_1/356.1_	0.023079	0.014
**PPG**						
	Average width	Session	8.27	_2/346.3_	0.00031	0.046
	Average peak	Session	5.03	_2/346.7_	0.006997	0.028
	HRV (sd)	Session	3.55	_2/347.1_	0.029858	0.020
**EOG**						
	Peak saccade velocity	Session	10.88	_2/4_	0.024103	0.845
	Saccade amplitude	Session	6.19	_2/348.7_	0.002288	0.034
**Eye-tracking**						
	Pupil diameter	Session	10.11	_2/4_	0.027267	0.835
	Fixation/saccade ratio	Session	3.97	_2/185.9_	0.02053	0.041
	Fixation/saccade ratio	Session^*^Condition	3.32	_2/174.2_	0.038615	0.037

fNIRS and EEG have been FDR-corrected. A lack of results for a factor (i.e., Condition, Interaction) indicates no significant results.

### 3.5 Situation Awareness

**Figure 7:**
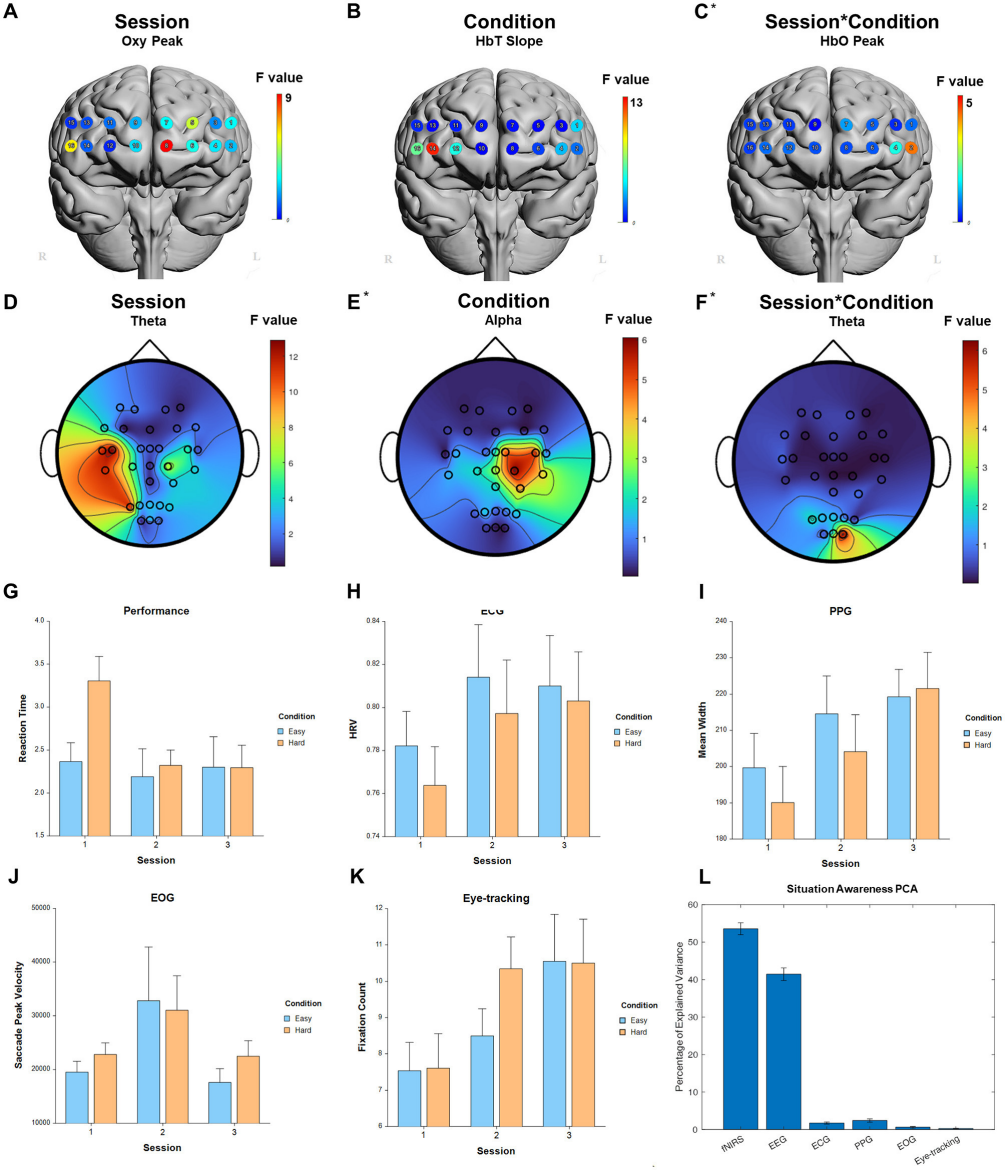
Selected results for Situation Awareness task. **(A–C)** LMM *F*-values for fNIRS across prefrontal cortex for the fixed factors of Session, Condition, and Interaction. **(D–F)** LMM *F*-values for EEG across whole brain for the fixed factors of Session, Condition, and Interaction. **(G)** Performance measure across session and condition. **(H)** ECG measure. **(I)** PPG measure. **(J)** EOG measure. **(K)** Eye-tracking measure. **(L)** PCA percentage of explained variance. *Significant before FDR correction.

For the fixed factor of session, significant measures included: 1 performance, 66 fNIRS (25 FDR-corrected), 129 EEG (95 FDR-corrected), 3 ECG, 5 PPG, 2 EOG, and 5 eye-tracking. For the fixed factor of condition, significant measures included: 3 performance, 21 fNIRS (3 FDR-corrected), and 15 EEG (0 FDR-corrected). For session and condition interaction, significant measures included: 1 performance, 4 fNIRS (0 FDR-corrected), 3 EEG (0 FDR-corrected), and 1 PPG. PCA analysis revealed an average of 18.7 PCs with eigenvalues >1, explaining 74.0% of the total variance. Of this, the following percentage of variance explained by each modality is: 54.2% fNIRS, 40.7% EEG, 1.8% ECG, 2.6% PPG, 0.6% EOG, and 0.2% eye-tracking. Selected results are presented in Figure 7, Table 5.

**Table 5:** Selected results of LMM processing for performance and each modality for Situation Awareness.

**Modality**	**Measure**	**Factor**	**F-ratio**		***p*-value**	**Partial η^2^**
**Performance**						
	Accuracy	Condition	6.17	_1/248_	0.013638	0.024
	Reaction time	Session	5.91	_2/248_	0.003107	0.045
	Reaction time	Condition	5.30	_1/248_	0.022133	0.021
	Reaction time	Session^*^Condition	3.64	_2/248_	0.027555	0.029
**fNIRS**						
	Oxy 08 peak	Session	9.22	_2/245.2_	0.000138	0.070
	HbR 15 peak	Session	11.00	_2/246.1_	0.000027	0.082
	HbT 11 peak	Session	13.44	_2/248_	0.000003	0.098
	HbT 14 slope	Condition	12.44	_1/205.2_	0.000517	0.057
	HbT 14 sum	Condition	9.15	_1/202.5_	0.002816	0.043
**EEG**						
	Theta FCC5h	Session	11.64	_2/142.7_	0.000021	0.140
	Theta FCC3	Session	12.93	_2/134.5_	0.000007	0.161
	Engagement FCC3	Session	19.52	_2/129.6_	0.000001	0.231
	Gamma FCC3	Session	26.58	_2/133.3_	0.000001	0.285
	Theta + Alpha/Beta + Alpha FCC3	Session	15.87	_2/136_	0.000001	0.189
**ECG**						
	HRV (rms)	Session	3.77	_2/224.7_	0.024524	0.032
	Low/High ratio	Session	3.80	_2/226.7_	0.023909	0.032
**PPG**						
	Heartrate	Session	4.47	_2/219.2_	0.01248	0.039
	Mean width	Session	5.69	_2/220.7_	0.0039	0.049
	Mean peak	Session	7.31	_2/221.8_	0.000845	0.062
	HRV (rms)	Session	4.85	_2/220.5_	0.008652	0.042
	HRV (sd)	Session^*^Condition	3.58	_2/218.1_	0.029594	0.032
**EOG**						
	Saccade peak velocity	Session	9.51	_2/4_	0.030176	0.826
	Saccade amplitude	Session	3.53	_2/222.4_	0.031049	0.031
**Eye-tracking**						
	Fixation count	Session	3.78	_2/135.6_	0.025296	0.053
	Mean fixation duration	Session	3.11	_2/138.7_	0.047501	0.043
	Saccade length	Session	3.17	_2/131.2_	0.045042	0.046

fNIRS and EEG have been FDR-corrected. A lack of results for a factor (i.e., Condition, Interaction) indicates no significant results.

### 3.6 Inhibitory Control

**Figure 8:**
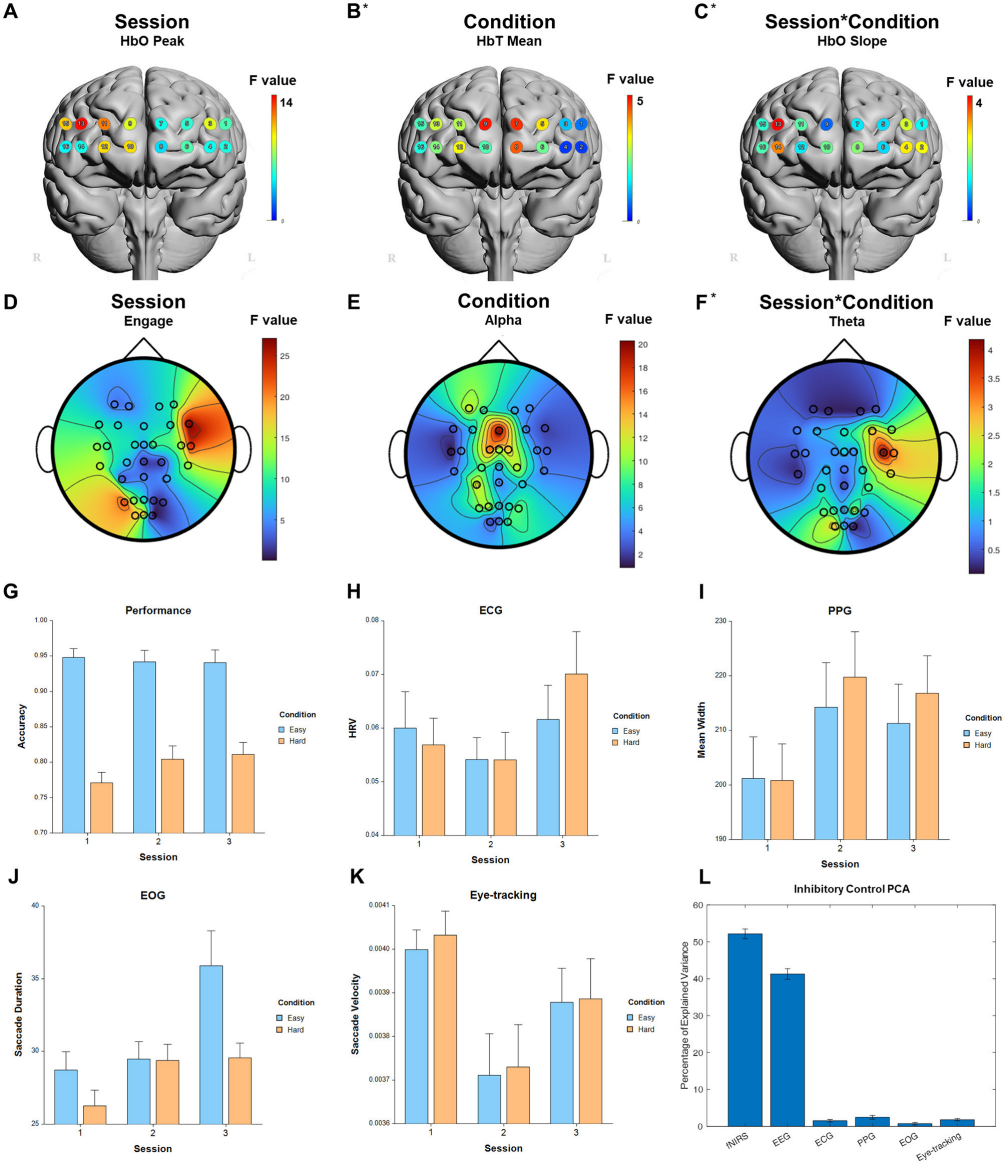
Selected results for Inhibitory Control task. **(A–C)** LMM *F*-values for fNIRS across prefrontal cortex for the fixed factors of Session, Condition, and Interaction. **(D–F)** LMM *F*-values for EEG across whole brain for the fixed factors of Session, Condition, and Interaction. **(G)** Performance measure across session and condition. **(H)** ECG measure. **(I)** PPG measure. **(J)** EOG measure. **(K)** Eye-tracking measure. **(L)** PCA percentage of explained variance. *Significant before FDR correction.

For the fixed factor of session, significant measures included: 3 performance, 81 fNIRS (58 FDR-corrected), 199 EEG (189 FDR-corrected), 2 ECG, 2 PPG, 3 EOG, and 2 eye-tracking. For the fixed factor of condition, significant measures included: 3 performance, 21 fNIRS (0 FDR-corrected), 40 EEG (19 FDR-corrected), and 1 EOG. For session and condition interaction, significant measures included: 10 fNIRS (0 FDR-corrected) and 6 EEG (0 FDR-corrected). PCA analysis revealed an average of 15.8 PCs with eigenvalues >1, explaining 74.0% of the total variance. Of this, the following percentage of variance explained by each modality is: 51.8% fNIRS, 41.9% EEG, 1.4% ECG, 2.2% PPG, 0.8% EOG, and 1.9% eye-tracking. Selected results are presented in Figure 8, Table 6.

**Table 6:** Selected results of LMM processing for performance and each modality for Inhibitory Control.

**Modality**	**Measure**	**Factor**	**F-ratio**		**p-value**	**Partial η2**
**Performance**						
	Reaction time	Session	4.33	_2/383.2_	0.013845	0.022
	Reaction time	Condition	160.49	_1/383.2_	0.000001	0.295
	Accuracy	Condition	236.44	_1/386_	0.000001	0.380
**fNIRS**						
	HbO 13 peak	Session	13.77	_2/384.3_	0.000002	0.067
	HbO 13 time-to-peak	Session	13.85	_2/384.1_	0.000002	0.067
	Oxy 03 time-to-peak	Session	13.94	_2/385_	0.000001	0.068
	Oxy 13 time-to-peak	Session	13.13	_2/384.1_	0.000003	0.064
	Oxy 15 time-to-peak	Session	14.89	_2/385_	0.000001	0.072
**EEG**						
	Alpha CCCP6h	Session	19.73	_2/179.1_	0.000001	0.181
	Engagement FFC6h	Session	27.21	_2/180.2_	0.000001	0.232
	Theta/Alpha FCC1h	Session	17.57	_2/199.1_	0.000001	0.150
	Alpha FFCz	Condition	20.34	_1/206.6_	0.000011	0.090
	Alpha CPP3h	Condition	12.66	_1/27_	0.001409	0.319
**ECG**						
	Heartrate	Session	4.67	_2/357.3_	0.009962	0.025
	HRV (sd)	Session	5.49	_2/358.8_	0.004485	0.030
**PPG**						
	Average width	Session	4.04	_2/352.3_	0.018457	0.022
	High frequency (abs)	Session	7.48	_2/4_	0.044553	0.789
**EOG**						
	Peak saccade velocity	Session	6.84	_2/339.6_	0.001227	0.039
	Saccade amplitude	Session	4.26	_2/341.1_	0.01482	0.024
	Saccade duration	Session	7.04	_2/348.8_	0.001005	0.039
	Saccade duration	Condition	7.65	_1/340.1_	0.005971	0.022
**Eye-tracking**						
	Pupil diameter	Session	12.27	_2/4_	0.019648	0.860
	Saccade velocity	Session	3.45	_2/205.3_	0.033705	0.032

fNIRS and EEG have been FDR-corrected. A lack of results for a factor (i.e. Condition, Interaction) indicates no significant results.

## 4 Discussion

The goal of this study was to measure the neural, physiological, and behavioral correlates of mental workload and learning across a mid-length (one month) longitudinal experiment in six distinct cognitive domains. In order to accomplish this, six different domains (Working Memory, Vigilance, Risk Assessment, Shifting Attention, Situational Awareness, and Inhibitory Control) were selected to be evaluated across four categories (Behavioral, Neural, Cardiac, Ocular), using six biometric approaches (EEG, fNIRS, ECG, PPG, EOG, eye-tracking). These composite measures were selected in part to identify and triage individual measures' suitability and compatibility for measurement of workload across and within different domains as well as the respective overlap between modalities.

Neurophysiological measurements in operational settings can be used to better understand and characterize the individual's experience, opening opportunities to enhance the overall approach. The aviation industry has particularly been at the forefront of investigating the neurophysiological measurements consistent with the neuroergonomic approach (Borghini et al., 2017; Callan and Dehais, 2019; van Weelden et al., 2022). In this study, we utilized real-world relevant adaptations of aviation related tasks for the cognitive domains.

Overall, we found that neural, ocular, and cardiac physiological measures showed consistent general sensitivity to participant experience and workload across tasks. The PCA analyses showed that over 90% of significant variation across all measures (corrected between subjects but not sessions or condition) were found in the fNIRS and EEG modalities. fNIRS measures, despite being fewer in number than EEG, accounted for a higher proportion of variability in every task. Looking at the results in Tables 1–6, it can be observed that although the F-values for EEG measures in the main factor of session were often higher than the corresponding fNIRS results, fNIRS provided significant information about the difference in brain activity relative to hard and easy task conditions, as well as some interactions. This outcome suggests that fNIRS and EEG in combination can provide a stronger picture of workload demands and the effects of task experience when used together.

This work was not the first time it was observed that fNIRS and EEG could provide useful measures of workload performance. In a series of multimodal studies, we tested that and incorporated multiple measures to potentially improve accuracy/performance of brain computer interfaces (Liu et al., 2013, 2015, 2017a,b; Sun et al., 2020). During an N-back working memory task with fNIRS, EEG, and cardiovascular measures, linear discriminant analysis was used to classify workload for each combination of fNIRS, EEG, and physiological signals (Liu et al., 2017b). While all three modalities showed the ability to classify workload levels, the fNIRS+EEG combination provided the best results, but the addition of heart rate and respiratory measures did not significantly improve classification in that study.

In this study, a similar marginal role was found for non-neural physiological measures. When examining the domains (Cardiac and Ocular), ~7% of remaining significant explained variance was accounted for by ECG and PPG measures of heart activity while Ocular measures of EOG and eye-tracking accounted for the remainder (~3%). For our chosen tasks and workload correlates, this suggests that heart activity is a better measure of monitoring expertise acquisition over training than Ocular measures. While this proves the strength of multimodal imaging, it does not dismiss the concept of incorporating measures of peripheral-physiological measures during real-time workload monitoring. While neural measures may be more sensitive to changes in task performance and conditions, peripheral measures are usually much more easily acquired and often at minimal cost. In addition, peripheral measures may often be used to contextualize and improve the sensitivity of neural measures and this topic is the subject of substantial research.

### 4.1 Behavioral measures

Across all tasks, we noted significant changes across task session and condition. In general, task performance improved across sessions and was decreased during harder conditions, as expected. In particular, the reaction time was the performance measure most likely to improve across sessions and across tasks. As the specific focus of this paper was on the contributions of neuro/physiological measures, we devote the rest of the discussion section to the biomedical modalities. A more detailed discussion of behavioral performance can be viewed in Supplementary material.

### 4.2 fNIRS measures

The Working Memory task displayed bilateral activation in the ventrolateral prefrontal cortex (vlPFC optodes 2 and 16) for the main effect of Session as well as a significant response to Condition in the right dlPFC (optode 15). This partially overlapped with a similar response for the Shifting Attention task in the right vlPFC. The Shifting Attention task in particular engaged memory of the locations of future targets to click on while focusing primary attention on the current target, explaining the potential overlap. Although Shifting Attention also showed differences between Condition, these differences were also observed broadly across the PFC. Working memory has been widely studied with functional neuroimaging, and has been shown induce task load related prefrontal activity with fNIRS (Ayaz et al., 2007, 2012; Fishburn et al., 2014; Herff et al., 2014; McKendrick et al., 2014; Fairclough et al., 2018; Chen et al., 2021) as well fMRI studies (Owen et al., 2005). Shifting Attention is also known to be correlated with activation in the dlPFC and vlPFC (Müller et al., 2014).

The Vigilance and Inhibitory Control tasks showed a similar pattern in results. The Vigilance task had the strongest sensitivity to session change in activation in the bilateral dorsolateral prefrontal cortex (dlPFC), as well as significant responses to Condition in the right ventromedial prefrontal cortex (vmPFC). Additionally, the Vigilance task showed a significant interaction in medial anterior PFC. The Inhibitory Control task showed overlap in the right dlPFC condition for the Session factor. The similarity in activation may both be due to the effects of learning to mitigate fatigue as both require high reflex reactions and hand-eye coordination over time. Vigilance, and its inversely related domain of fatigue, are well studied in neuroscience, as they are particularly important in sustained focus tasks such as driving and piloting (Paxion et al., 2014; Dehais et al., 2018). Inhibitory Control as well requires similar levels of continuous, sustained focus and the ability to react in time to specific targets (Rodrigo et al., 2014).

Both the Risk Assessment and Situation Awareness tasks had activation changes in the left ventromedial prefrontal cortex, which makes sense as both are using high level assessments of an entire situation as it changes over time, requiring the updating of mental models in response to an evolving environment. The Risk Assessment task was also sensitive to Condition in the left dlPFC, whereas the Situation Awareness task was sensitive to Condition in the right vlPFC. These categories of tasks may be able to be distinguished over time using fNIRS. Numerous previous studies into risk taking attitudes have used fNIRS, EEG, PET, and fMRI to distinguish not just between skill levels, but entirely different strategies that may utilize different brain areas and networks (Compagne et al., 2023). Situation awareness of the environment and periphery, both in the lab and outside of it in the real world, can benefit from knowing specific brain areas to target, thus lowering the amount of potentially impeding equipment (McKendrick et al., 2016; Ismail and Karwowski, 2020).

Overall, the fNIRS models showed the strongest sensitivity to Session for all tasks with a preference for the bilateral PFC for Working Memory and Vigilance tasks, and specifically the right-lateral PFC for Shifting Attention and Inhibitory Control tasks. Risk Assessment and Situation Awareness tasks instead engaged the left medial PFC. These regions partially overlapped with significant regions for Condition, however it is the differences between the main factors of Session and Condition that allow researchers to distinguish between skill acquisition over time and the reaction to different difficulty levels. In the future, it would be best to include more than two conditions for each task to better understand the brain's reaction over a range instead of just binary high and low workload. Similarly, using more graded performance factors would allow for a more realistic and nuanced understanding of cognitive workload, which can then be applied in the field with operators at work or in training to actively monitor workload in real time.

### 4.3 EEG measures

The Working Memory task, Shifting Attention task, and Situation Awareness tasks all showed broad significant responses in the theta band in response to the main effect of Session. Changes in both the alpha and theta bands are inherent to memory and cognition, usually being inversely related to one another, but task-specific demands may cause one to change more than the other, leading to significance in either one band or the ratio between them (Klimesch, 1999). On the other hand, Condition for Working Memory task was predominantly responsive in the alpha band in the central parietal and right parietal occipital lobes, whereas both Shifting Attention and Situation Awareness showed a response to Condition in the right central and central parietal regions. Shifting Attention also showed more widespread changes in the theta/alpha band ratio whereas the Situational Awareness task localized these changes primarily in the right hemisphere. In general, these power band changes are known to occur in the parietal regions of the cortex in response to difficulty and changes over time in tasks involving the memory (Sauseng et al., 2005; Gulbinaite et al., 2014).

The Vigilance task was broadly responsive to the main effect of Session and Condition in the alpha band as well as the delta band for Condition. In addition to the focus for the task normally being seen in the alpha band, the delta band is correlated with fatigue over time, especially in driver studies (Lal and Craig, 2001, 2002; Stikic et al., 2011).

The engagement ratio showed a broad sensitivity to Session for the Risk Assessment and Inhibitory Control tasks. In addition, Inhibitory Control also showed a particularly strong response to Condition in the bilateral central alpha band from the frontal to occipital regions, as the ability to inhibit a natural response is related to risk-related decision making. The engagement ratio takes into account the alpha, theta, and beta band powers together, providing correlates both related to fatigue (as is important in Inhibitory Control) and attention needed to correctly assess the changing risk levels of a task with both short term and long term goals (Prinzel et al., 2000; Berka et al., 2007).

While EEG measures were widely significant across the brain for changes in the main effect of Session, not every task elicited a significant main effect of Condition with the EEG data after FDR correction (only Vigilance and Inhibitory Control). Overall, the alpha band was the most sensitive to changes in difficulty, and real world applications may focus on this and the theta band, over the central and parietal regions of the cortex, to try and use a limited number of electrodes that are faster and easier to set up as compared to whole brain recordings for more neuroergonomic applications (Mehta and Parasuraman, 2013; Ismail and Karwowski, 2020; Longo et al., 2022).

### 4.4 Cardiac measures

In both ECG and PPG based cardiac monitoring modalities, we found the primary significant factor to be Session, with very few measures found to have a significant effect on Condition and Interaction. Across all six tasks, ECG measures of heartrate variability and low frequency components were found to have a linear relationship with session, decreasing over time as expertise was gained. This matches well with previous studies making use of ECG in workload (Shaffer and Ginsberg, 2017; Marchand et al., 2021). With PPG, pulse width was most commonly the highest significant measure with respect to sessions and displayed an inverse linear relationship with time. Interestingly, we also observed an inverted-U shape curve for the pulse peak measure in the hard condition of the Shifting Attention task, reminiscent of the Yerkes-Dodson performance-workload curve (Yerkes and Dodson, 1908; Sibi et al., 2016). Overall, heart rate variability was found to be the most consistent workload correlate. Cardiac measures are the most commonly used physiological measure for mental workload from the literature (Charles and Nixon, 2019), and thus are a good candidate to investigate for learning/adaptation over time.

### 4.5 Ocular measures

The primary significant factor was Session in both EOG and Eye-tracking based ocular measures. Saccade measurements calculated using EOG over all tasks revealed that the mean duration and amplitude while on task generally show an inverse linear relationship with workload, increasing as workload over time decreases, similar to previous findings (Di Stasi et al., 2010). The timing of the increases seen in Inhibitory Control, with a larger increase early on in the hard condition and an increase later for easy, may also provide more information about the level of skill and how learning on different difficulties may result in alternate patterns. More interestingly, the peak saccade velocity showed an inverse-U shape over the three sessions in several tasks, aligning with the shape of moving from one learning plateau to the next. Eye-tracking measures showed a consistent response for saccade velocity as well as an increase in fixation count, both of which are known workload correlates (Ahlstrom and Friedman-Berg, 2006; Faller et al., 2019).

Of all the ocular measures, pupil diameter appeared to provide the most information about workload over time with nearly every task showing significance for Session over time with a smaller diameter indicating increased skill. Using wearable eye-tracking devices, this ocular measure can be taken anywhere, especially since eye gaze is not directly involved in pupillometry (Marchand et al., 2021). Caution must be taken, however, because the environment, particularly the amount of light, can have a big effect on pupil size. Relative measurements taken over a task would provide the most useful information about workload changes within a given period of time.

### 4.6 Limitations and future directions

A key finding from this study was that physiological measures and neural measures in particular show a strong and generalizable responsiveness to task demand and participant task experience. However, these measures were not necessarily able to effectively distinguish between performance demands due to different cognitive domains. Some sensitive measures showed an inverse relationship between workload and task experience, suggesting that physiological measures are good trackers of overall learning over time, but they are less sensitive to specific difficulties associated with individual cognitive domains. However, some of this variability may be due to the usage of fixed difficulty levels instead of more continuous measures of task demand which are better representative of real-word task performance.

Sensitivity to individual cognitive domains could be improved with increased participant pool size and increased repetition of specific tasks. Another limitation of the task battery format was the relatively short length of each individual task. The choice to examine participants across multiple cognitive domains necessarily reduced the number of trials each participant could perform. As a result, this reduced the experimental power within each cognitive domain. Future work may include the use of multi-domain tasks with overlapping cognitive requirements as a means of compressing task battery performance or the use of multi-tasking for individual tasks across different domains.

Some additional variation in the results may be due to the non-uniform sampling of task performance. During protocol development, we had to account for the ability of participants to maintain focus throughout the entire experimental session and encourage them to return once each week for a month. Setting up six different neuroimaging and physiological monitoring modalities can put significant physical strain on participants, and so we were limited in how long each session could be. Accordingly, the protocol was developed so that not every task was performed during every experimental session. Although the randomized task assignment was non-uniform, it may however be more realistic and representative of typical task practice as individuals do not necessarily train under entirely fixed intervals. Furthermore, this did not appear to result in worsened task performance as participants reliably improved across sessions.

We found some overlap in the active brain areas in our neuroimaging analyses, but for this publication we have only analyzed the sensitivity of neural measures within each separate task. In future analyses, we plan to not just look across modalities but across tasks as well to search for both unique and shared markers of different types of induced workload. We can also use this dataset to search for predictors of future performance, similarly to our previously published results for the Inhibitory Control task (Ayaz et al., 2019). This may allow for training to be further optimized by determining learners who may require more or less attention on various tasks. Future studies can also apply this approach to other domains such as clinical studies for neurological and psychiatric conditions as a tool for triage and diagnosis.

## 5 Conclusion

We developed a highly multimodal non-invasive framework of neuroimaging and physiological monitoring modalities to assess the effect of training and expertise development during a longitudinal task battery targeting six fundamental cognitive domains. Six different cognitive domains (Working Memory, Vigilance, Risk Assessment, Shifting Attention, Situational Awareness, and Inhibitory Control) were selected to be evaluated across four categories (Behavioral, Neural, Cardiac, Ocular) using six biometric approaches (fNIRS, EEG, ECG, PPG, EOG, Eye-tracking). This novel, comprehensive study revealed the most sensitive measures to workload and skill across both time and task difficulty effects for prefrontal fNIRS, whole head EEG, two heart activity measures, and two eye activity measures. Several measures within modalities were found to be consistent across tasks, but the neuroimaging modalities in particular revealed useful differences between cognitive domains as well. This study lays the groundwork for future multimodal studies in addition to the analysis of more complex experiments in the neuroergonomic domain of studying the brain and body at work in the real world.

## Data availability statement

The original contributions presented in the study are included in the article/Supplementary material, further inquiries can be directed to the corresponding author.

## Ethics statement

The studies involving humans were approved by Institutional Review Board of Drexel University. The studies were conducted in accordance with the local legislation and institutional requirements. The participants provided their written informed consent to participate in this study.

## Author contributions

JM: Data curation, Formal analysis, Investigation, Methodology, Validation, Visualization, Writing—original draft. AC: Data curation, Formal analysis, Investigation, Methodology, Software, Validation, Writing—review & editing. AK: Investigation, Methodology, Writing—review & editing. MZ: Conceptualization, Funding acquisition, Investigation, Methodology, Project administration, Resources, Supervision, Validation, Writing—review & editing. HA: Conceptualization, Formal analysis, Funding acquisition, Investigation, Methodology, Project administration, Resources, Supervision, Visualization, Writing—review & editing.
