# Supplementary material for: Mental workload assessment by monitoring brain, heart, and eye with six biomedical modalities during six cognitive tasks
Source: Front Neuroergon. 2024 Mar 12;5:1345507. doi: 10.3389/fnrgo.2024.1345507 (PMC10963413; doi:10.3389/fnrgo.2024.1345507)
Supplement: Supplementary file 2 [file Data_Sheet_1.docx]

Supplementary Material for

Mental Workload Assessment by Monitoring Brain, Heart, and Eye with Six Biomedical Modalities During Six Cognitive Tasks

Jesse Mark^1^, Adrian Curtin^1^, Amanda Kraft^2^, Matthias Ziegler^2^, Hasan Ayaz^1,3,4,5,6,7*^

^1^School of Biomedical Engineering, Science, and Health Systems, Drexel University, Philadelphia PA, United States

^2^Advanced Technology Laboratories, Lockheed Martin, Arlington, VA, United States

^3^ Department of Psychological and Brain Sciences, College of Arts and Sciences, Drexel University, Philadelphia, PA, United States

^4^ Drexel Solutions Institute, Drexel University, Philadelphia, PA, United States,

^5^ A. J. Drexel Autism Institute, Drexel University, Philadelphia, PA, United States,

^6^ Department of Family and Community Health, University of Pennsylvania, Philadelphia, PA, United States,

^7^ Center for Injury Research and Prevention, Children’s Hospital of Philadelphia, Philadelphia, PA, United States

## Additional Discussion of Behavioral Measures

Across all tasks, we found the most significant changes with the main factor of session. This factor indicates skill improvements based on experience regardless of task difficulty. In particular, the reaction time performance measure improved across sessions for every task. Only Inhibitory Control and Shifting Attention had additional performance improvements over sessions. For Inhibitory Control, the d-prime, or sensitivity to discern inhibit signals from true targets, improved over time. In Shifting Attention, the accuracy, mouse velocity, and total trial time improved over sessions.

For five of the six tasks, we developed two distinct difficulty levels intended to induce states of low and high workload. Only for Vigilance, which was based on the continuous performance task, were we required to make a concession to the different levels. To reiterate, because this task was a five-minute nonstop attention task designed to induce fatigue, we split the block into eight equal-length segments, the first four representing low workload and the latter four representing high workload. Each task had more significant performance differences for the main factor of condition than for session; this is notable because the neuroimaging and physiological monitoring were more sensitive to changes over time.

The Working Memory task differed in mean position error and reaction time between conditions. The difference between low and high workload was memorizing either five or seven targets and marking their locations with the mouse within a set amount of time. The error for the high difficulty rose as expected, whereas the reaction time was lower. This may have been due to subjects attempting to click the mouse faster before they forgot all seven target locations, which in turn decreased their accuracy.

In Vigilance, we found that the accuracy, true positive rate, d-prime, and reaction times worsened with condition (i.e., over time from the beginning to the end of the task). As fatigue sets in, participants become more disengaged and distracted, leading to a decrease in performance. Due to the nature of the task battery requiring short individual task times, the full effects of vigilance and fatigue may not have been elucidated with this experiment and may need further testing.

The Risk Assessment task prompted participants to maximize the total earnings of a collection task. In the low workload condition, earnings increased linearly and there was a lower chance of losing everything, whereas in the high workload condition earnings increased exponentially but there was a greater chance of losing the collected points. These distinct conditions made considerable differences in the number of collections, earnings, and losses per trial. This may be an inherent function of the task design itself, which was based on the BART and adapted for our purposes. Future versions could spread out the risk more such that a single unpredictable mistake would not end the trial or lessen the increase in earnings to be between linear and exponential.

Shifting Attention, or the trail-making test, did have a high number of significant performance measures for all factors. However, this was mainly due to the skill ceiling of the low workload condition. Clicking numbered circles in order may be seen to induce minimal workload, as the original task is primarily used to assess cognitive impairment, which our healthy participants did not suffer from. In assessing this cognitive domain in the future, we would recommend an alternative task or one that increases the difficulty even further, adding three or four sequential lists instead of only numbers and letters.

The Situation Awareness task was split into two parts per trial: the video portion and the question portion. During the video part, participants checked either one or three planes flying a predetermined path and compared their actions to gauges monitoring speed, direction, and fuel for discrepancies. We saw significant differences between conditions in the accuracy and response times, and this task format can easily be expanded to include two, four, or more planes, or more or fewer gauges to make more gradations of difficulty.

The Inhibitory Control task also displayed an effect of difficulty condition on the accuracy, sensitivity, and reaction time. However, because we split the workload conditions into inhibit only (hard condition) and ignore only (easy condition), we could not study the effects of mixing true targets, ignore targets, and inhibit targets in a single combined block. This would be a more realistic situation, and workload could be modulated via the proportion of each type of stimulus.

We did not find many significant session and condition interactions for performance measures. Shifting Attention has already been discussed as an effect of the skill ceiling for the low workload condition. The only other interaction was reaction time in Situation Awareness, which can be seen as a higher value in session 1 for the hard condition (Figure 7). After acclimating to the task demands, participants began responding to each question within the same amount of time regardless of difficulty.

## Entire list of mixed model results

## Table S1: All tasks complete linear mixed models results.

Entire list is included in a separately attached excel spreadsheet file.

## Explanation of terms in the spreadsheet:

Risk task performance measures:

RSK_SucClick: The number of clicks on targets that did not crash.

RSK_Clicks: The total number of clicks on targets, including crashes.

RSK_Earnings: The number of points gained.

RSK_Crashed: The number of times a crash target was clicked.

RSK_meanRT: The mean amount of time between clicks.

RSK_stdRT: The standard deviation of time between clicks.

Situation awareness task performance measures:

SA_Accuracy: The percentage of correct answers to the questions.

SA_meanRT: The mean reaction time to answer the questions.

SA_stdRT: The standard deviation of the reaction time.

Vigilance/attention task performance measures:

VIG_Accuracy: The total percentage of correct responses, both ignore and click.

VIG_TruePos: The true positive percentage of correct responses to target stimuli.

VIG_TrueNeg: The true negative percentage of correct non-responses to non-targets.

VIG_Dprime: The sensitivity index calculated as the normalized difference between true positive and false negative (response to non-target).

VIG_meanRT: The mean reaction time.

VIG_stdRT: The standard deviation of the reaction time.

Working memory task performance measures:

WM_meanPosErr: The mean distance between response location and true stimuli target, calculated from the closest target to response with no overlap.

WM_stdPosErr: The standard deviation of response distance.

WM_meanRT: The mean reaction time.

WM_stdRT: The standard deviation of the reaction time.

Inhibitory control task performance measures:

IC_Accuracy: The total percentage of correct responses, both inhibit and click.

IC_Dprime: The sensitivity index calculated as the normalized difference between true positive and false negative (response to non-target).

IC_meanRT: The mean reaction time to targets.

IC_stdRT: The standard deviation of the reaction time.

Shifting attention/Trail-making task performance measures:

TMT_Correct: The percentage of correct responses compared to total clicks.

TMT_meanRT: The mean reaction time.

TMT_stdRT: The standard deviation of the reaction time.

TMT_meanVel: The mean velocity of cursor movement.

TMT_TrialTime: The length of each trial.

ECG:

ECG_heartRate: The heart rate.

ECG_sdISI: The standard deviation of heart rate.

ECG_rmsISI: The root mean square of heart rate.

ECG_LFabs: The absolute low frequency power.

ECG_HFabs: The absolute high frequency power.

ECG_LFrel: The relative low frequency power.

ECG_HFrel: The absolute high frequency power.

ECG_LFHFratio: The ratio between low and high frequency power.

EOG:

EOG_vPeak: The peak saccade velocity.

EOG_Duration: The mean saccade duration.

EOG_Amplitude: The mean saccade amplitude.

PPG:

PPG_heartRate: The heart rate.

PPG_aveWidth: The mean pulse width.

PPG_avePeak: The mean pulse peak amplitude.

PPG_sdISI: The standard deviation of heart rate.

PPG_rmsISI: The root mean square of heart rate.

PPG_LFabs: The absolute low frequency power.

PPG_HFabs: The absolute high frequency power.

PPG_LFrel: The relative low frequency power.

PPG_HFrel: The absolute high frequency power.

PPG_LFHFratio: The ratio between low and high frequency power.

EEG: Relative power spectra for five power bands Delta, Theta, Alpha, Beta, Gamma, and the four ratios of Engagement (Beta/(Alpha+Theta)), Theta/Alpha, Theta/Beta, and TABA ((Theta+Alpha)/(Beta+Alpha)).

Eye-tracking:

SacVel: Eye-tracking mean saccade velocity.

PupDia: Eye-tracking mean pupil diameter.

Fixate: Eye-tracking number of fixations.

MeanFix: Eye-tracking mean fixation duration.

MedFix: Eye-tracking median fixation duration.

FixRate: Eye-tracking rate of fixations per second.

FixSacRatio: Eye-tracking ratio of fixations to saccades.

SacLength: Eye-tracking mean saccade distance.

fNIRS: Four measures were processed in five ways.

HbO: Relative oxygenated hemoglobin change.

HbR: Relative deoxygenated hemoglobin change.

HbT: Total sum of oxygenated and deoxygenated hemoglobin.

Oxy: Difference between oxygenated and deoxygenated hemoglobin.

Mean: The mean change of marker during the block.

Slope: The slope of the first order linear regression best fit line during the block.

Time2Peak (TimeP): The time until the peak absolute value during the block.

Peak: The peak absolute value during the block.

Sum: The integrated sum of marker changes during the block.
